# Unraveling haplotype errors in the DFNA33 locus

**DOI:** 10.3389/fgene.2023.1214736

**Published:** 2023-08-21

**Authors:** Barbara Vona, Sabrina Regele, Aboulfazl Rad, Nicola Strenzke, Justin A. Pater, Katrin Neumann, Marc Sturm, Tobias B. Haack, Antoinette G. Am Zehnhoff-Dinnesen

**Affiliations:** ^1^ Tübingen Hearing Research Centre, Department of Otolaryngology, Head and Neck Surgery, Eberhard Karls University Tübingen, Tübingen, Germany; ^2^ Institute of Human Genetics, University Medical Center Göttingen, Göttingen, Germany; ^3^ Institute for Auditory Neuroscience and InnerEarLab, University Medical Center Göttingen, Göttingen, Germany; ^4^ Department of Phoniatrics and Pedaudiology, University Hospital Münster, University of Münster, Münster, Germany; ^5^ Auditory Systems Physiology Group, Department of Otolaryngology and Institute for Auditory Neuroscience, University Medical Center Göttingen, Göttingen, Germany; ^6^ Dana-Farber Cancer Institute, Harvard Medical School, Boston, MA, United States; ^7^ Health Sciences Centre, Faculty of Medicine, Memorial University, St. John’s, NL, Canada; ^8^ Institute of Medical Genetics and Applied Genomics, University of Tübingen, Tübingen, Germany; ^9^ Centre for Rare Diseases, University of Tübingen, Tübingen, Germany

**Keywords:** *ATP11A*, DFNA33, genome sequencing, haplotype analysis, hereditary hearing loss, unresolved deafness loci

## Abstract

Genetic heterogeneity makes it difficult to identify the causal genes for hearing loss. Studies from previous decades have mapped numerous genetic loci, providing critical supporting evidence for gene discovery studies. Despite widespread sequencing accessibility, many historically mapped loci remain without a causal gene. The DFNA33 locus was mapped in 2009 and coincidentally contains *ATP11A*, a gene recently associated with autosomal dominant hearing loss and auditory neuropathy type 2. In a rare opportunity, we genome-sequenced a member of the original family to determine whether the DFNA33 locus may also be assigned to *ATP11A*. We identified a deep intronic variant in *ATP11A* that showed evidence of functionally normal splicing. Furthermore, we re-assessed haplotypes from the originally published DFNA33 family and identified two double recombination events and one triple recombination event in the pedigree, a highly unlikely occurrence, especially at this scale. This brief research report also serves as a call to the community to revisit families who have previously been involved in gene mapping studies, provide closure, and resolve these historical loci.

## 1 Introduction

Meiotic recombination (also called crossing over) underlies genomic diversity and maintains fidelity during chromosome segregation. Although each chromosome generally presents at least one crossover (Sun et al., 2005), the recombination rate is not fixed but rather influenced by intrinsic (for example, genetic background (Koehler et al., 2002)) and extrinsic (such as age and environment (Williamson et al., 1970; Rose and Baillie, 1979; Tanzi et al., 1992)) factors and has a non-uniform distribution, with evidence of recombination “hot” and “cold spots” (Kong et al., 2002). Furthermore, the recombination rate and chromosome size are strongly correlated, with smaller chromosomes having higher recombination rates (Sun et al., 2005; Farré et al., 2013). Recombination frequencies are a measure of the genetic linkage and are essential for constructing linkage maps. Genes that are closer together have a greater chance of being inherited together due to a lower likelihood of undergoing fewer recombination events. Double crossovers are regarded as rare events, with the vast majority of double recombinations being the result of genotyping errors (Housworth and Stahl, 2003).

By definition, linkage is an occurrence in which adjacent genes tend to be inherited together (Wang et al., 2017). Linkage analysis requires genome-wide genotyping of families and is used to investigate how traits are segregated. Specifically, it can estimate whether phenotypes have a tendency to be inherited together, indicating the closeness of genes responsible for those traits in the genome. Linkage analysis of families segregating a Mendelian condition, such as autosomal dominant hearing impairment, aims to uncover few statistically significant linkage intervals, significantly reducing the number of candidate regions that are investigated in targeted sequencing. These studies have proven enormously successful for gene identification and have been re-defined and transformed in several ways, following the widespread emergence and application of disruptive high-throughput sequencing technologies (Teare and Santibañez Koref, 2014; Bamshad et al., 2019).

Hereditary hearing impairment is among the most genetically heterogeneous disorders observed in humans, making gene mapping and identification a comparatively enormous task (Wright et al., 2018). Family participation has been paramount to advancing the field. These families have been valuable for historical gene mapping efforts, following the uninformative screening of select known genes. The Hereditary Hearing Loss Homepage (https://hereditaryhearingloss.org/) has diligently documented mapped loci and regularly updates content with new genes as they are discovered. Despite the advent of widely accessible sequencing technologies, many of these families have been lost to follow up for a variety of reasons. As a result, it is a reasonable assumption that many of these loci will remain without a causal gene assigned. Furthermore, in spite of the success of mapping deafness loci and cost-effective genome sequencing technologies, the identification of causal genes is not always straightforward, even in families that are large and, therefore, informative by providing more information about meiotic recombination events. This is additionally reflected in the large number of families who have participated in historical linkage studies but remain without a causal gene identified. At least 20 autosomal dominant (DFNA) loci have been mapped (Hereditary Hearing Loss Homepage) that remain without a causal gene assigned. The majority of them were mapped before the widespread use of high-throughput sequencing. There are also several examples of independent investigators discovering the same gene under a pre-assigned DFN locus. For example, the DFNA20 and DFNA26 loci merged as DFNA20/26 when two teams discovered *ACTG1* as a gene responsible for autosomal dominant hearing loss for these asynchronously mapped loci (van Wijk et al., 2003; Zhu et al., 2003). Among the remaining loci with an unknown causal gene is DFNA33 that was published in 2009 through the ascertainment of a large, multi-generational German family, segregating progressive sensorineural non-syndromic hearing loss with a variable post-lingual onset (Bönsch et al., 2009). This brief research report describes follow-up genome sequencing analysis in light of the recent discovery of *ATP11A*, a gene that is responsible for autosomal dominant non-syndromic hearing loss (DFNA84) (Pater et al., 2022) and auditory neuropathy (AUNA2) (Chepurwar et al., 2022), which coincidentally also overlaps with the DFNA33 genomic coordinates at chr13q34.

## 2 Methods

### 2.1 Participant recruitment and audiometry

Through extensive re-recruitment efforts, an affected individual from the originally published DFNA33 family (Bönsch et al., 2009) was re-ascertained as part of a large diverse-population rare disease study (ethics commission approval number 197/2019BO1). The pedigree was re-evaluated and re-drawn.

The affected individual underwent a complete ear, nose, and throat examination that included binocular ear microscopy and external ear inspection. Pure-tone audiometry was performed according to current standards to determine hearing thresholds at routinely measured frequencies (0.25, 0.5, 1, 2, 4, 6, and 8 kHz). Both air and bone conduction thresholds were measured. Tympanometry was performed to measure tympanic membrane compliance and middle ear pressure.

### 2.2 Whole-genome sequencing, bioinformatics filtering, and variant classification

Whole genome sequencing was performed on genomic DNA extracted from peripheral blood using standard protocols, as previously described (Weisschuh et al., 2020). The sequencing library was prepared with the TruSeq DNA PCR-free protocol (Illumina, San Diego, USA) for subsequent sequencing as 2 × 150 bp paired-end reads on a NovaSeq 6000 platform (Illumina, San Diego, USA). Bioinformatic processing of raw read data, annotation, and variant calling was performed using the megSAP pipeline (https://github.com/imgag/megSAP) developed at the Institute of Medical Genetics and Applied Genomics, University Hospital Tübingen, Germany, and includes an in-house genome database called the NGS Database (NGSD), which consists data on more than 3,000 individuals to ascertain sequencing artifacts and variant frequencies. Visualization was performed using the Integrative Genomics Viewer.

Bioinformatics filters were used to support the analysis of variants, following autosomal dominant and recessive modes of inheritance, as described previously (Falb et al., 2023). This included analysis of sequence variants and copy number variations in 298 known hearing loss-associated genes (Supplementary Table S1). Allele-based filters selecting variants with an allele frequency (including sub-populations) ≤ 1%, variants present in ≤20 other individuals in NGSD, and those previously annotated as pathogenic/likely pathogenic in HGMD and ClinVar yielded a short list of heterozygous variants in these hearing loss-associated genes. Minor allele frequencies were derived from gnomAD, 1000 Genomes Project, ExAC, dbSNP, and NGSD. The Alamut Visual (Interactive Biosoftware, SOPHiA GENETICS, Rouen, France) splice window that includes splice predictions from SpliceSiteFinder-like, MaxEntScan, NNSPLICE, GeneSplicer, and ESEfinder, as well as RESCUE-ESE predictors, was used to assess the effect of variants on splicing.

Variant classification applied the American College of Medical Genetics and Genomics (ACMG) guidelines that are adapted for hereditary hearing loss (Oza et al., 2018) and referenced the Deafness Variation Database (Azaiez et al., 2018). The classification was assisted through the use of the public version of Varsome (Kopanos et al., 2019).

### 2.3 *In vitro* splicing analysis

A region spanning *ATP11A* exons 8 to 10 was PCR-amplified from the genomic DNA of the patient and a healthy control using gene-specific primers with a *Xho*I restriction site (*ATP11A* Ex8-10 forward: 5′-aat​tct​cga​gAA​AAT​CAC​CGA​AGC​CAT​GAG-3′) and a *Bam*HI restriction site (*ATP11A* Ex8-10 reverse: 5′-att​gga​tcc​ATG​GTG​AGA​GGA​GCT​GTT​GG-3′). The 5,014-bp amplicon was ligated into a multiple cloning site between native exons A and B in a linearized pSPL3 exon-trapping vector. The vector was transformed into DH5α competent cells (NEB 5-alpha, New England Biolabs) and plated overnight. The wild-type and mutant-containing vector sequences were confirmed by Sanger sequencing and transfected into HEK 293T cells using the FuGENE Transfection Reagent (Promega). Total RNA was prepared from 24-h post-transfected cells using an miRNeasy Mini Kit (QIAGEN), and reverse transcription was carried out using a High-Capacity RNA-to-cDNA Kit (Applied Biosystems). Amplified fragments were visualized on 1.5% agarose gel and, subsequently, Sanger sequenced.

## 3 Results

### 3.1 Audiological characterization

**FIGURE 1:**
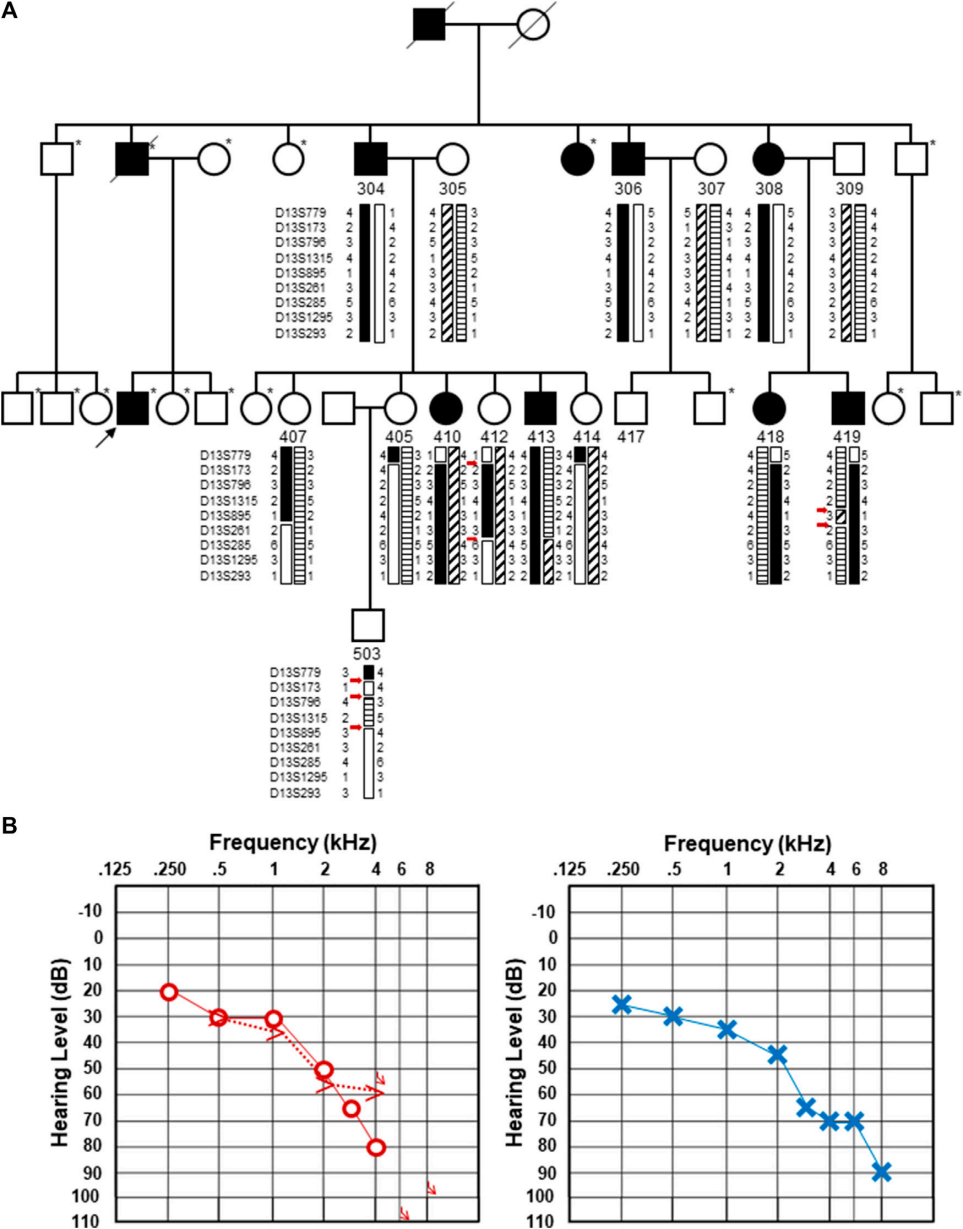
Updated pedigree and clinical information from the individual whose genome was subjected to sequencing. **(A)**. Updated pedigree with an emphasis on the third generation. The individual who was sequenced is marked with a black arrow. Individuals who were not included in the Bönsch et al. (2009) publication are marked with an asterisk to the upper right of each pedigree symbol. Those included in the original pedigree are noted with their designated number from Bönsch et al. (2009). Haplotypes originally described in Bönsch et al. (2009) are included with the double and triple recombination events marked with red arrows. It should be noted that individual 407 was originally denoted as a male individual in the original pedigree. Upon re-review, an error was identified, and this has been updated and corrected as a female individual. **(B)**. Pure-tone audiograms from the individual who was subjected to genome sequencing. Pure-tone audiogram from the right (left) and left (right) ears at the age of 66.9 years. Air conduction thresholds in the dB hearing level for the right and left ears are represented with circles and crosses, respectively. Bone conduction measurements are available for the right ear and shown by >.

The affected individual, who was not in the original DFNA33 report, is a direct descendent of a hearing-impaired father who had four affected siblings, three of whom were previously described (Bönsch et al., 2009) (Figure 1A). The individual reported progressive, high-frequency sensorineural hearing impairment without subjective vertigo and tinnitus. The individual's thresholds mirror the average thresholds of individuals previously described in their family (Bönsch et al., 2009) up to 2 kHz, where the individual shows steeply increasing thresholds to severe-to-profound hearing loss at 8 kHz in the left ear and no thresholds at 6 and 8 kHz in the right ear. An audiogram at the age of 66.9 years is shown in Figure 1B. A tympanogram showed normal bilateral results with regular tympanic membrane compliance. Upon comparison of the audiogram with the hearing of men in the same age group (ISO, 2017), the hearing thresholds at all measured frequencies were found to be significantly lower than the expected hearing threshold for this age group.

### 3.2 Genome sequencing, bioinformatics analysis, and the *in vitro* splicing assay of a deep intronic variant

Whole-genome sequencing resulted in an average 51.7 × coverage of the genome. An analysis of sequence variants and copy number variations in 289 known hearing loss-associated genes (Supplementary Table S1) excluded a putative pathogenic genetic aberration. These variants, shown in Supplementary Table S2, were excluded based on criteria such as their presence in a homozygous state in other presumably normal hearing individuals in NGSD, low *in silico* pathogenicity prediction scores, no predicted effect on splicing, or an obvious clinical mismatch.

**FIGURE 2:**
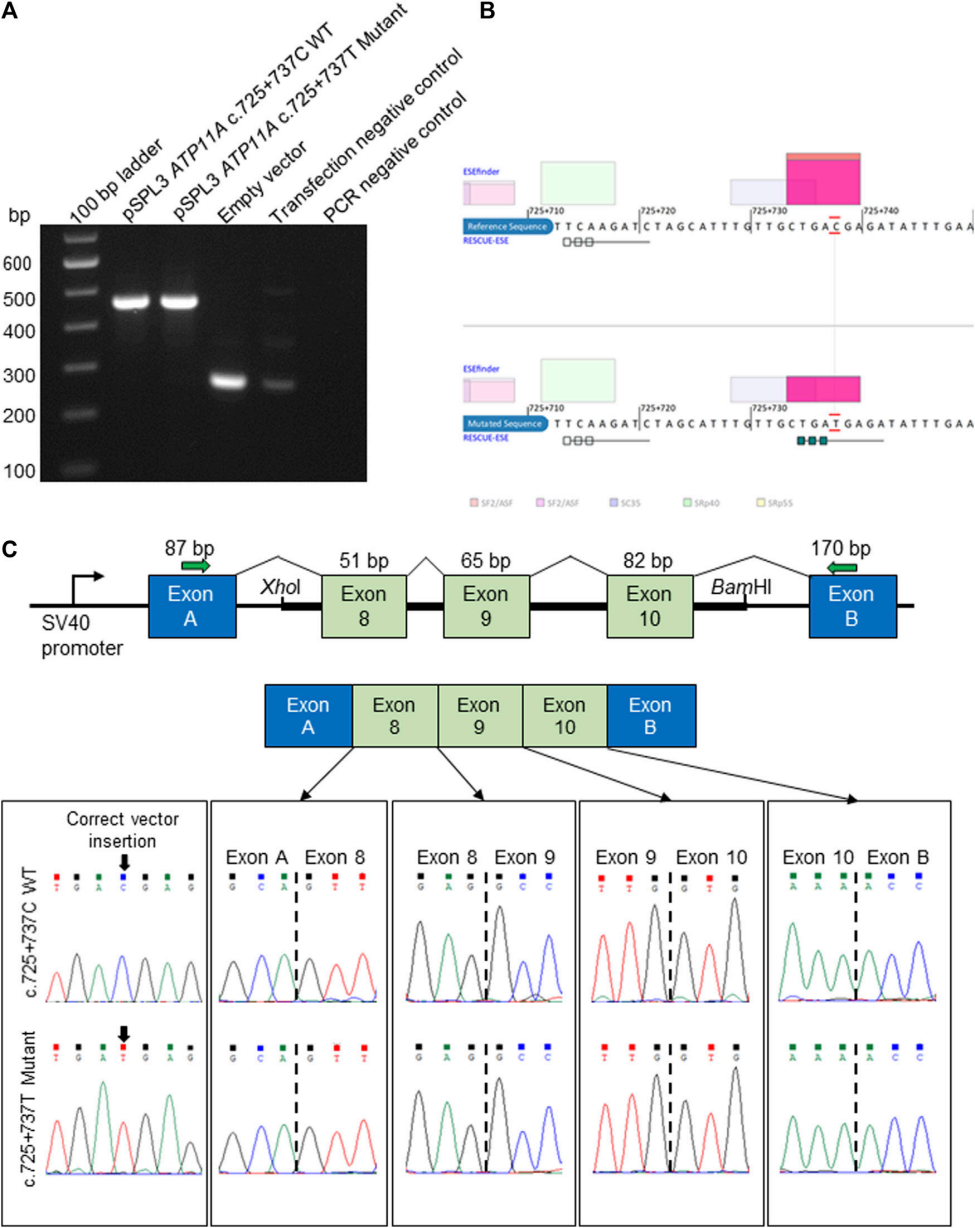
*In vitro* splicing assay and *in silico* prediction of the *ATP11A* c.725 + 737C>T deep intronic variant. **(A)**. Electrophoretic visualization of cDNA RT-PCR products amplified from the constructs, following transfection into HEK 293T cells. Amplicons were resolved on 1.5% agarose gel. Wild-type splicing yields a 455-bp amplicon and is composed of exons A and B (vector) and exons 8–10 that were amplified from the patient and control DNA samples. **(B)** Analysis with ESEfinder and RESCUE-ESE reveals the splicing sequence landscape for the wild-type (upper panel) and mutated (lower panel) human sequence at c.725 + 737. The nucleotide at the c.725 + 737 position is outlined in red. ESE hits are displayed above and below each sequence. The green boxes represent RESCUE-ESE hexamers. The c.725 + 737C>T variant is predicted to induce an ESE hexamer that is shown by the string of green boxes in the bottom sub-panel. **(C)** The vector construct of the *in vitro* splicing assay illustrates the wild-type or mutant amplicons inserted between exons A and B of the pSPL3 vector with a normal splicing result (upper and mid panels). The lower panel shows the sequence of each exon–exon junction for wild-type and mutant constructs, revealing an identical result.

Following the initial hypothesis that *ATP11A* may be the gene for the DFNA33 locus and the growing clinical and functional evidence asserting ATP11A is an essential protein for a normal functioning auditory system, our analysis then focused on the previously mapped DFNA33 locus coordinates (GRCh37:110,300,001-115,169,878), as well as *ATP11A* (Supplementary Table S3), and uncovered variants that were either identified in other in-house patients with presumed normal hearing or were likely benign based on bioinformatics assessment. Only one deep intronic variant in *ATP11A* intron 8 (chr13:113421269C>G (GRCh37), ENST00000375630:c.725 + 737C>T) was predicted via *in silico* tools to activate exonic splice enhancers. This variant was tested with an *in vitro* splicing assay using established protocols (Rad et al., 2021; Vona et al., 2021) and yielded a functionally normal result (Figure 2). Taken together, the variant is classified as likely benign (PM2_Supporting, BP4_Supporting, and BS3_Strong; −4 points (likely benign point range: −6 to −1)). On this basis, we conclude that following short-read genome sequencing, a method employed for the identification of the first described *ATP11A* variant (Pater et al., 2022), we present evidence that does not support *ATP11A* as being the gene for the DFNA33 locus and acknowledge that we have been extremely fortunate to re-recruit an individual from the DFNA33 family.

### 3.3 Haplotype review

Upon close inspection, we noted that the haplotypes that were used to define the DFNA33 boundaries of the disease interval in the original Bönsch et al. paper show several unlikely events (Bönsch et al., 2009, shown in Figure 1A for clarity). These include three recombination events in a small chromosomal interval in individual 503 and two recombination events in a relatively short interval in individuals 412 and 419. The fine mapping and recapitulation of haplotypes seem improbable and may be due to genotyping or other errors. Due to this observation, the DFNA33 disease interval may be inaccurate. Therefore, we performed genome-wide analysis of variants that did not yield an obvious candidate and informative result for follow-up and thus required additional family members in order to significantly narrow variants.

## 4 Discussion

Although a conventional understanding of recombination rates approximates about one crossover per homologous pair of chromosomes, it varies dramatically on a number of different scales and shows non-uniform distribution. However, mapping recombination patterns have uncovered chromosomal regions enriched for hotspots that are separated from regions that do not appear to recombine. The deCODE genetic map created chromosomal genetic maps that also included sex-averaged recombination rates (Kong et al., 2002). Analysis of the chr13q34 region with these data does not suggest the DFNA33 locus is a recombination hotspot (data not shown).

Closely adjacent double or triple recombination events involving phasing errors are suggestive of genotyping errors (Housworth and Stahl, 2003). Although crossing over may appear random, it is tightly regulated and occurs at a low frequency due to factors that prevent excessive recombination and the disruption of favorable genetic combinations (Séguéla-Arnaud et al., 2015). Although we should have noted the unlikely double and triple recombination events in individuals 412, 419, and 503 previously, performing genome sequencing and functional analysis of a candidate splice variant was important for excluding a newly associated disease gene in the previously mapped significant interval.

With increased access to advanced sequencing technologies, the continuous follow-up of undiagnosed families with historical linkage is essential for the discernment of correct DFN-locus assignment. Our study highlights the necessity and potential to refine understanding of the number of total unresolved loci through a simple review of historical loci for such unlikely events. A problematic bottleneck will be re-contacting and re-establishing contact with the original families as maintaining connections to families over decades can be challenging. Considering the size of this unresolved family, although unlikely, we cannot exclude multi-locus heterogeneity or the presence of phenocopies. Furthermore, it was unfortunate that we could not recruit the entire family for repeat linkage analysis and genome sequencing of additional affected individuals to increase the chances of uncovering the causal genetic variant. The most convincing case for excluding DFNA33 altogether would be through the identification of a causal variant, either within the DFNA33 locus or elsewhere in the genome, which is a limiting factor of our work. Using a short-read genome-sequencing approach, we cannot exclude epigenetic modifications or short-inverted duplications. Nonetheless, re-assessment of haplotypes combined with new genome sequencing data can provide some value in better understanding unsolved DFNA loci.

## Data Availability

The datasets presented in this study can be found in online repositories. The names of the repository/repositories and accession number(s) can be found at: https://databases.lovd.nl/shared/individuals/00435034, #00435034.
